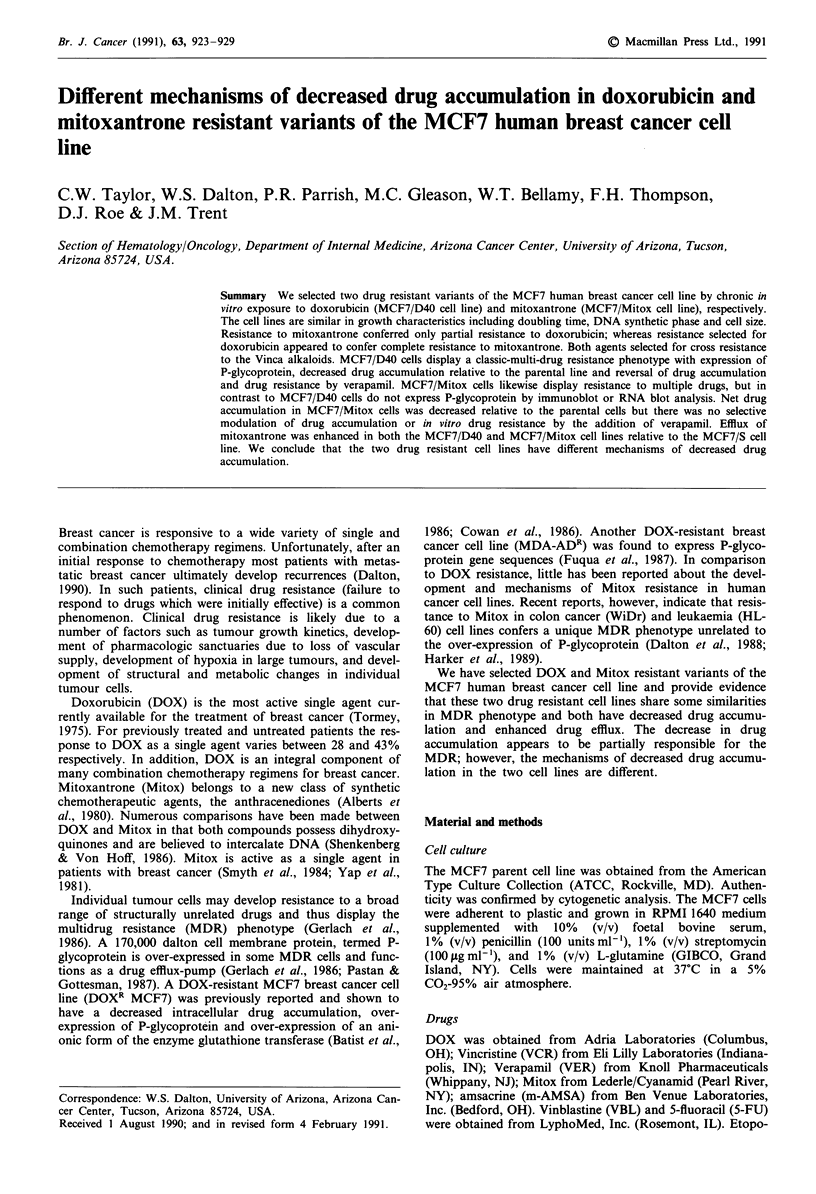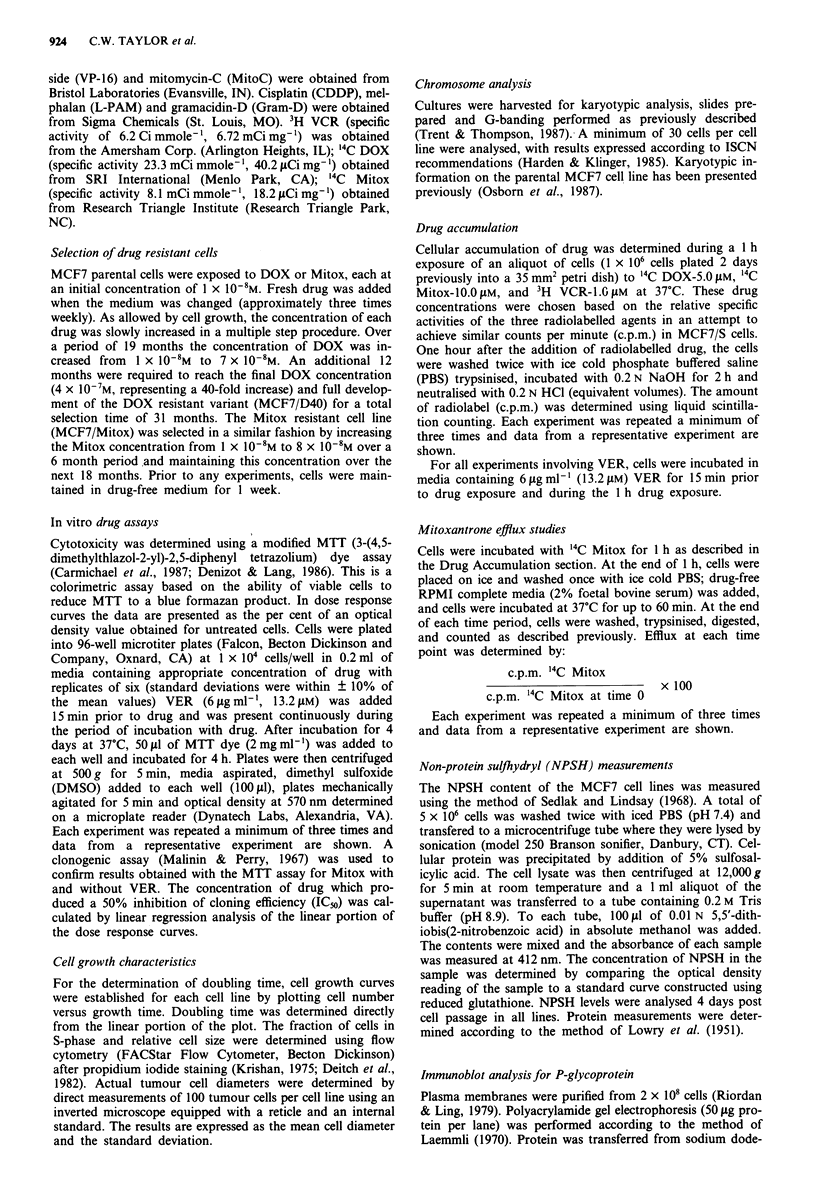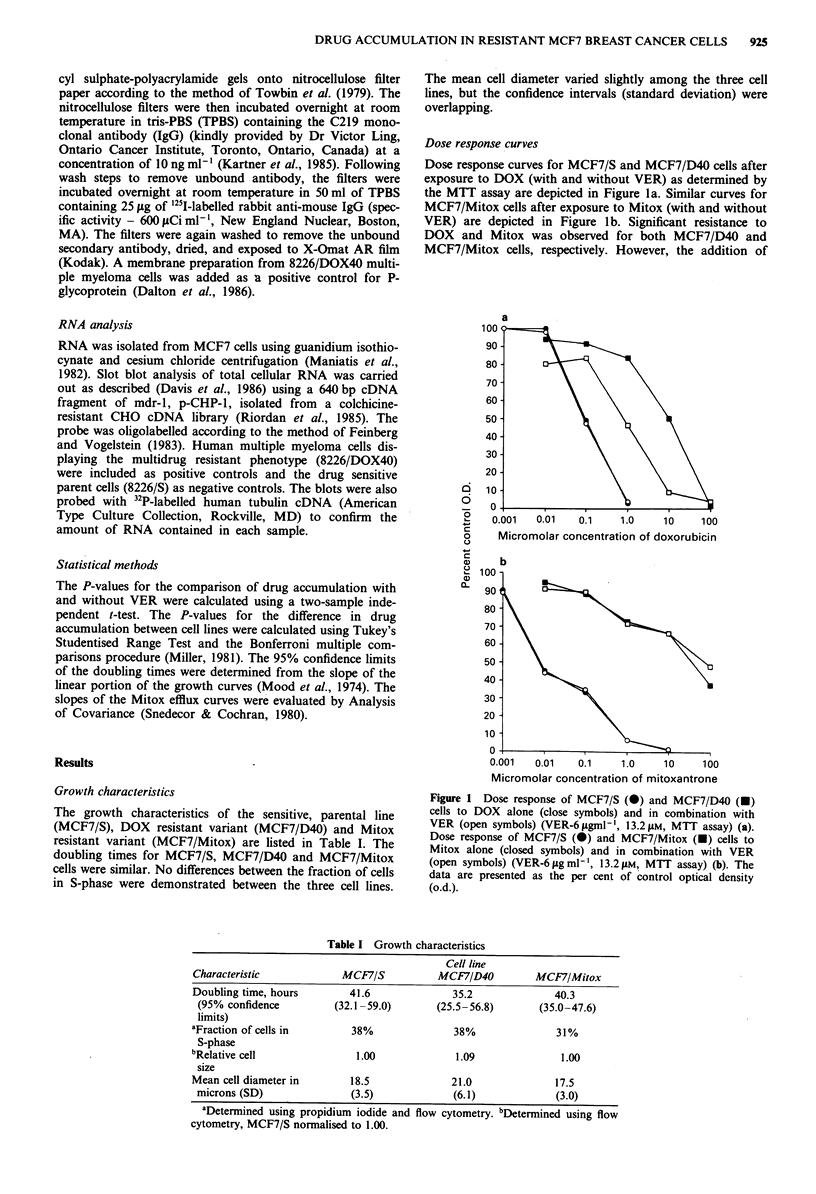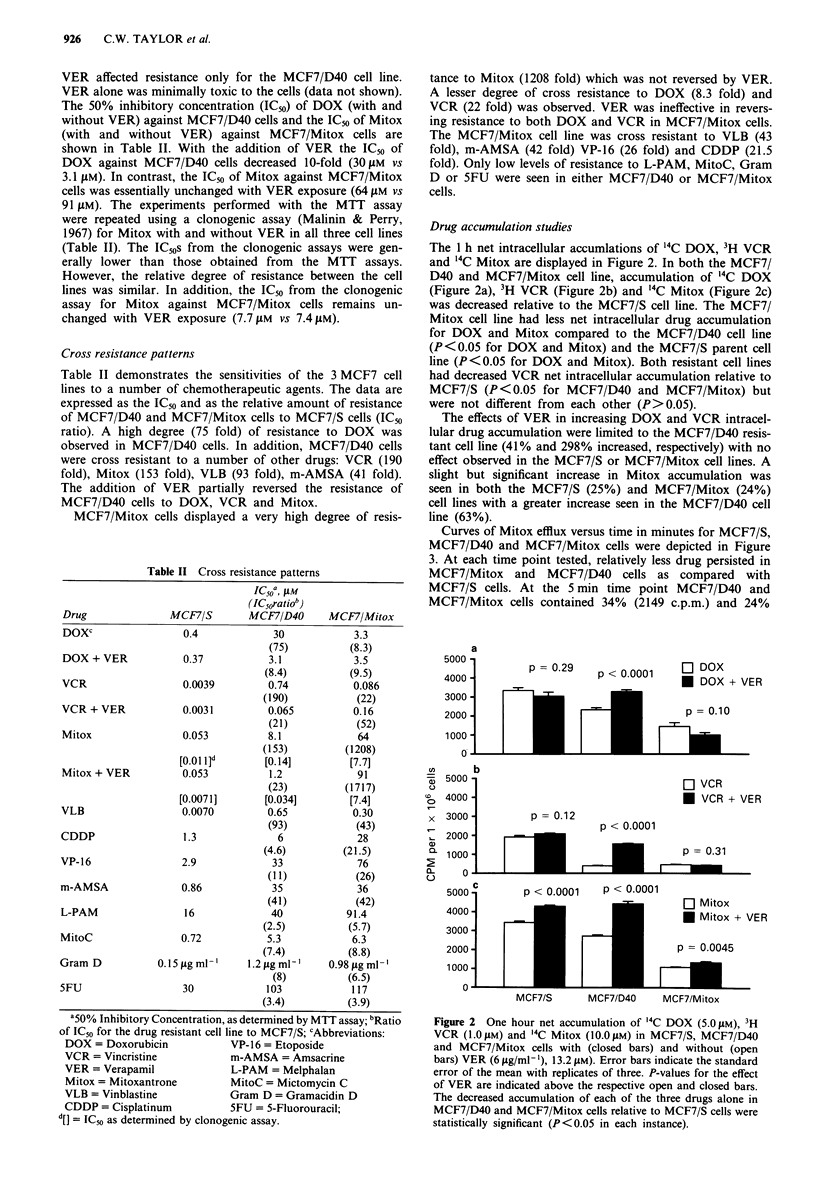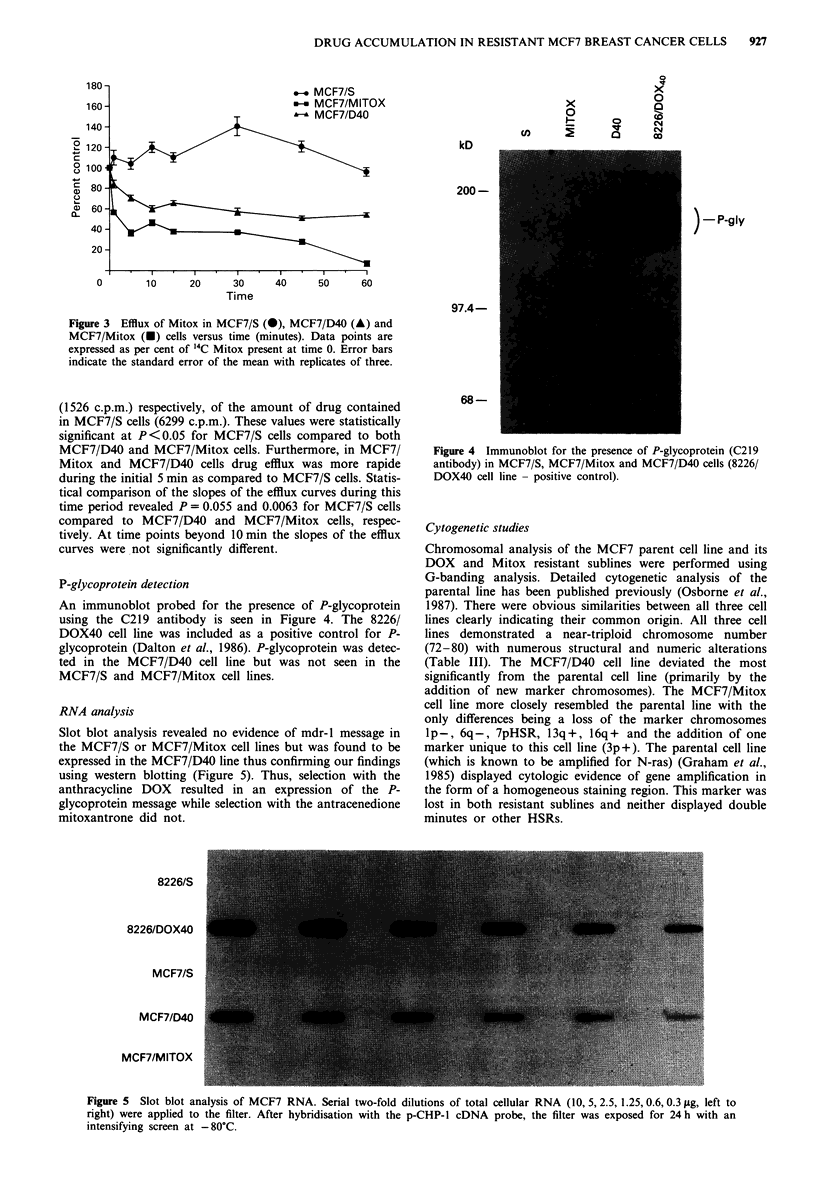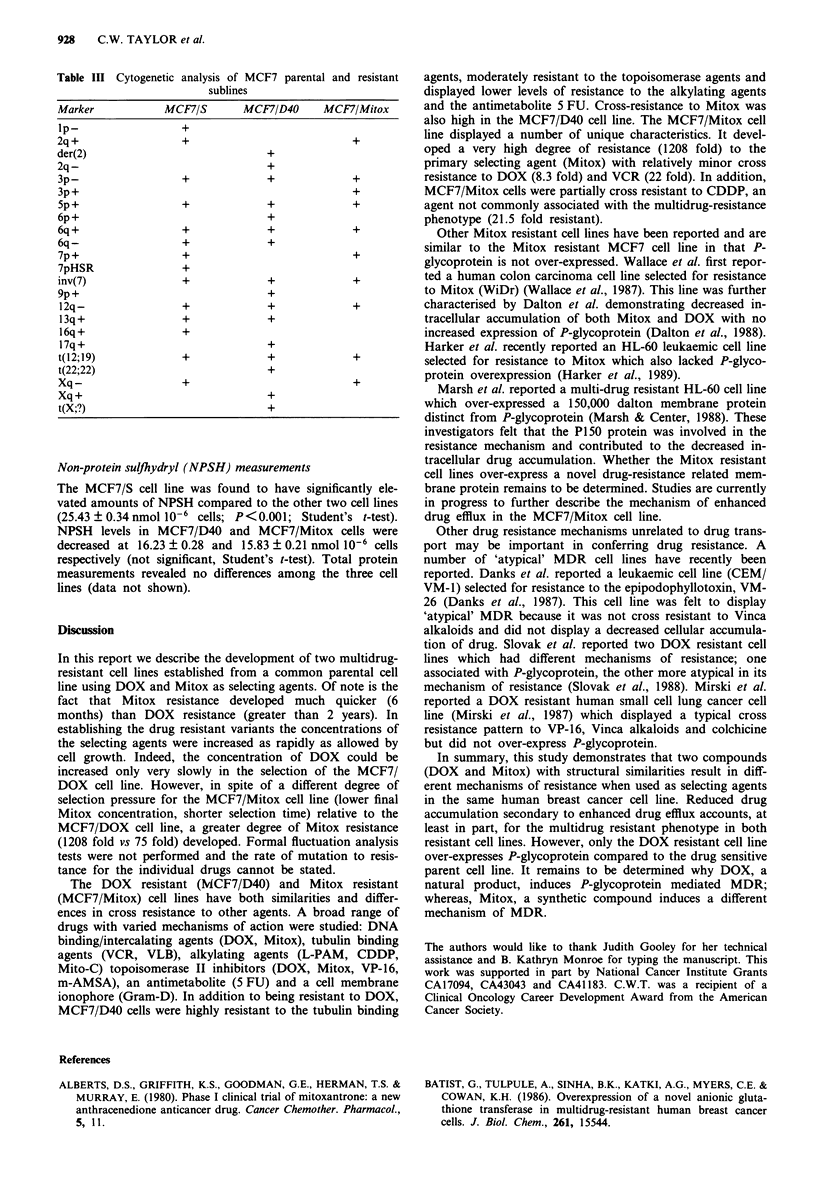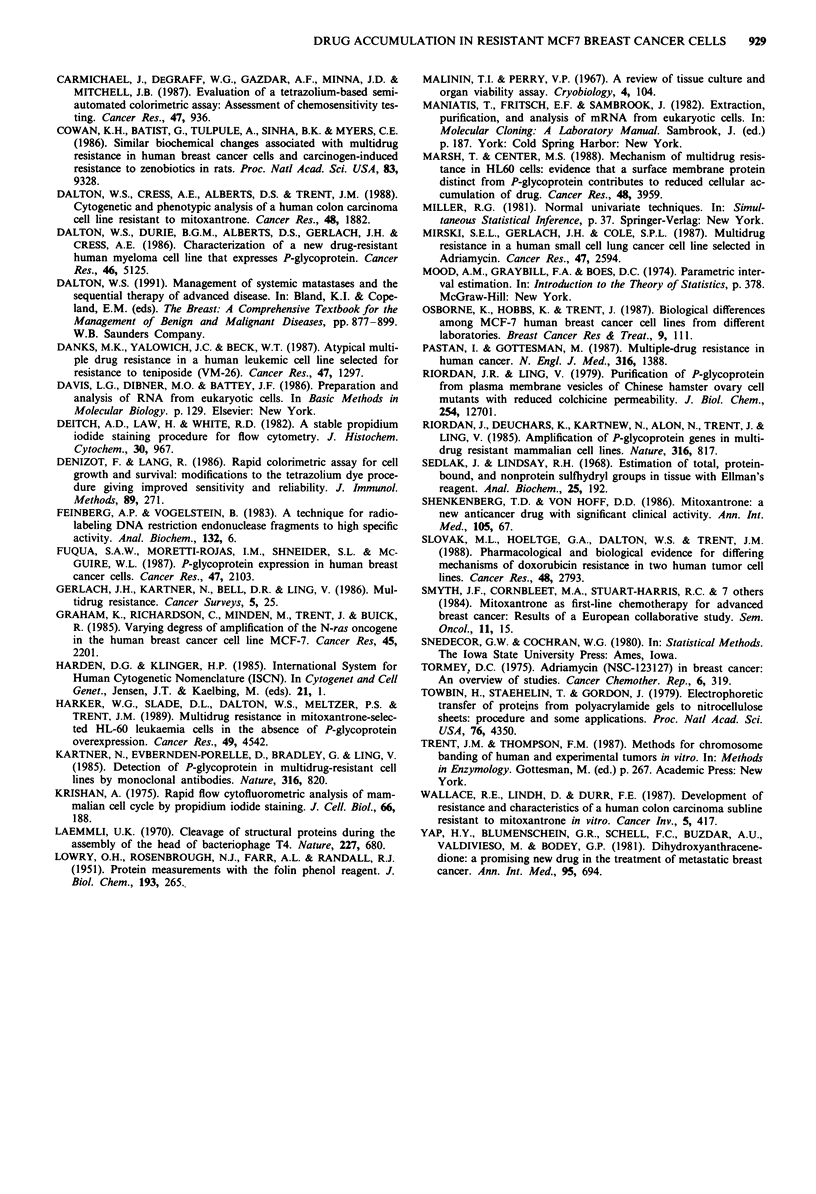# Different mechanisms of decreased drug accumulation in doxorubicin and mitoxantrone resistant variants of the MCF7 human breast cancer cell line.

**DOI:** 10.1038/bjc.1991.202

**Published:** 1991-06

**Authors:** C. W. Taylor, W. S. Dalton, P. R. Parrish, M. C. Gleason, W. T. Bellamy, F. H. Thompson, D. J. Roe, J. M. Trent

**Affiliations:** Department of Internal Medicine, Arizona Cancer Center, University of Arizona, Tucson 85724.

## Abstract

**Images:**


Br. J. Cancer (1991), 63, 923-929 © Macmillan Press Ltd., 1991

### Different mechanisms of decreased drug accumulation in doxorubicin and mitoxantrone resistant variants of the MCF7 human breast cancer cell line

C.W. Taylor, W.S. Dalton, P.R. Parrish, M.C. Gleason, W.T. Bellamy, F.H. Thompson, D.J. Roe & J.M. Trent

*Section of Hematology/Oncology, Department of Internal Medicine, Arizona Cancer Center, University of Arizona, Tucson, Arizona 85724, USA.*

**Summary** We selected two drug resistant variants of the MCF7 human breast cancer cell line by chronic *in vitro* exposure to doxorubicin (MCF7/D40 cell line) and mitoxantrone (MCF7/Mitox cell line), respectively. The cell lines are similar in growth characteristics including doubling time, DNA synthetic phase and cell size. Resistance to mitoxantrone conferred only partial resistance to doxorubicin; whereas resistance selected for doxorubicin appeared to confer complete resistance to mitoxantrone. Both agents selected for cross resistance to the Vinca alkaloids. MCF7/D40 cells display a classic-multi-drug resistance phenotype with expression of P-glycoprotein, decreased drug accumulation relative to the parental line and reversal of drug accumulation and drug resistance by verapamil. MCF7/Mitox cells likewise display resistance to multiple drugs, but in contrast to MCF7/D40 cells do not express P-glycoprotein by immunoblot or RNA blot analysis. Net drug accumulation in MCF7/Mitox cells was decreased relative to the parental cells but there was no selective modulation of drug accumulation or *in vitro* drug resistance by the addition of verapamil. Efflux of mitoxantrone was enhanced in both the MCF7/D40 and MCF7/Mitox cell lines relative to the MCF7/S cell line. We conclude that the two drug resistant cell lines have different mechanisms of decreased drug accumulation.

Breast cancer is responsive to a wide variety of single and combination chemotherapy regimens. Unfortunately, after an initial response to chemotherapy most patients with metastatic breast cancer ultimately develop recurrences (Dalton, 1990). In such patients, clinical drug resistance (failure to respond to drugs which were initially effective) is a common phenomenon. Clinical drug resistance is likely due to a number of factors such as tumour growth kinetics, development of pharmacologic sanctuaries due to loss of vascular supply, development of hypoxia in large tumours, and development of structural and metabolic changes in individual tumour cells.

Doxorubicin (DOX) is the most active single agent currently available for the treatment of breast cancer (Tormey, 1975). For previously treated and untreated patients the response to DOX as a single agent varies between 28 and 43% respectively. In addition, DOX is an integral component of many combination chemotherapy regimens for breast cancer. Mitoxantrone (Mitox) belongs to a new class of synthetic chemotherapeutic agents, the anthracenediones (Alberts *et al.*, 1980). Numerous comparisons have been made between DOX and Mitox in that both compounds possess dihydroxyquinones and are believed to intercalate DNA (Shenkenberg & Von Hoff, 1986). Mitox is active as a single agent in patients with breast cancer (Smyth *et al.*, 1984; Yap *et al.*, 1981).

Individual tumour cells may develop resistance to a broad range of structurally unrelated drugs and thus display the multidrug resistance (MDR) phenotype (Gerlach *et al.*, 1986). A 170,000 dalton cell membrane protein, termed P-glycoprotein is over-expressed in some MDR cells and functions as a drug efflux-pump (Gerlach *et al.*, 1986; Pastan & Gottesman, 1987). A DOX-resistant MCF7 breast cancer cell line ($DOX^R$ MCF7) was previously reported and shown to have a decreased intracellular drug accumulation, over-expression of P-glycoprotein and over-expression of an anionic form of the enzyme glutathione transferase (Batist *et al.*, 1986; Cowan *et al.*, 1986). Another DOX-resistant breast cancer cell line ($MDA\text{-}AD^R$) was found to express P-glycoprotein gene sequences (Fuqua *et al.*, 1987). In comparison to DOX resistance, little has been reported about the development and mechanisms of Mitox resistance in human cancer cell lines. Recent reports, however, indicate that resistance to Mitox in colon cancer (WiDr) and leukaemia (HL-60) cell lines confers a unique MDR phenotype unrelated to the over-expression of P-glycoprotein (Dalton *et al.*, 1988; Harker *et al.*, 1989).

We have selected DOX and Mitox resistant variants of the MCF7 human breast cancer cell line and provide evidence that these two drug resistant cell lines share some similarities in MDR phenotype and both have decreased drug accumulation and enhanced drug efflux. The decrease in drug accumulation appears to be partially responsible for the MDR; however, the mechanisms of decreased drug accumulation in the two cell lines are different.

### Material and methods

### *Cell culture*

The MCF7 parent cell line was obtained from the American Type Culture Collection (ATCC, Rockville, MD). Authenticity was confirmed by cytogenetic analysis. The MCF7 cells were adherent to plastic and grown in RPMI 1640 medium supplemented with 10% (v/v) foetal bovine serum, 1% (v/v) penicillin (100 units $ml^{-1}$), 1% (v/v) streptomycin (100 $\mu g\ ml^{-1}$), and 1% (v/v) L-glutamine (GIBCO, Grand Island, NY). Cells were maintained at 37°C in a 5% $CO_2$-95% air atmosphere.

### *Drugs*

DOX was obtained from Adria Laboratories (Columbus, OH); Vincristine (VCR) from Eli Lilly Laboratories (Indianapolis, IN); Verapamil (VER) from Knoll Pharmaceuticals (Whippany, NJ); Mitox from Lederle/Cyanamid (Pearl River, NY); amsacrine (m-AMSA) from Ben Venue Laboratories, Inc. (Bedford, OH). Vinblastine (VBL) and 5-fluoracil (5-FU) were obtained from LyphoMed, Inc. (Rosemont, IL). Etopo-

Correspondence: W.S. Dalton, University of Arizona, Arizona Cancer Center, Tucson, Arizona 85724, USA.
Received 1 August 1990; and in revised form 4 February 1991.

side (VP-16) and mitomycin-C (MitoC) were obtained from Bristol Laboratories (Evansville, IN). Cisplatin (CDDP), melphalan (L-PAM) and gramacidin-D (Gram-D) were obtained from Sigma Chemicals (St. Louis, MO). $^{3}$H VCR (specific activity of 6.2 Ci mmole$^{-1}$, 6.72 mCi mg$^{-1}$) was obtained from the Amersham Corp. (Arlington Heights, IL); $^{14}$C DOX (specific activity 23.3 mCi mmole$^{-1}$, 40.2 $\mu$Ci mg$^{-1}$) obtained from SRI International (Menlo Park, CA); $^{14}$C Mitox (specific activity 8.1 mCi mmole$^{-1}$, 18.2 $\mu$Ci mg$^{-1}$) obtained from Research Triangle Institute (Research Triangle Park, NC).

*Selection of drug resistant cells*

MCF7 parental cells were exposed to DOX or Mitox, each at an initial concentration of $1 \times 10^{-8}$M. Fresh drug was added when the medium was changed (approximately three times weekly). As allowed by cell growth, the concentration of each drug was slowly increased in a multiple step procedure. Over a period of 19 months the concentration of DOX was increased from $1 \times 10^{-8}$M to $7 \times 10^{-8}$M. An additional 12 months were required to reach the final DOX concentration ($4 \times 10^{-7}$M, representing a 40-fold increase) and full development of the DOX resistant variant (MCF7/D40) for a total selection time of 31 months. The Mitox resistant cell line (MCF7/Mitox) was selected in a similar fashion by increasing the Mitox concentration from $1 \times 10^{-8}$M to $8 \times 10^{-8}$M over a 6 month period and maintaining this concentration over the next 18 months. Prior to any experiments, cells were maintained in drug-free medium for 1 week.

In vitro *drug assays*

Cytotoxicity was determined using a modified MTT (3-(4,5-dimethylthlazol-2-yl)-2,5-diphenyl tetrazolium) dye assay (Carmichael et al., 1987; Denizot & Lang, 1986). This is a colorimetric assay based on the ability of viable cells to reduce MTT to a blue formazan product. In dose response curves the data are presented as the per cent of an optical density value obtained for untreated cells. Cells were plated into 96-well microtiter plates (Falcon, Becton Dickinson and Company, Oxnard, CA) at $1 \times 10^{4}$ cells/well in 0.2 ml of media containing appropriate concentration of drug with replicates of six (standard deviations were within $\pm$ 10% of the mean values) VER (6 $\mu$g ml$^{-1}$, 13.2 $\mu$M) was added 15 min prior to drug and was present continuously during the period of incubation with drug. After incubation for 4 days at 37°C, 50 $\mu$l of MTT dye (2 mg ml$^{-1}$) was added to each well and incubated for 4 h. Plates were then centrifuged at 500 *g* for 5 min, media aspirated, dimethyl sulfoxide (DMSO) added to each well (100 $\mu$l), plates mechanically agitated for 5 min and optical density at 570 nm determined on a microplate reader (Dynatech Labs, Alexandria, VA). Each experiment was repeated a minimum of three times and data from a representative experiment are shown. A clonogenic assay (Malinin & Perry, 1967) was used to confirm results obtained with the MTT assay for Mitox with and without VER. The concentration of drug which produced a 50% inhibition of cloning efficiency ($IC_{50}$) was calculated by linear regression analysis of the linear portion of the dose response curves.

*Cell growth characteristics*

For the determination of doubling time, cell growth curves were established for each cell line by plotting cell number versus growth time. Doubling time was determined directly from the linear portion of the plot. The fraction of cells in S-phase and relative cell size were determined using flow cytometry (FACStar Flow Cytometer, Becton Dickinson) after propidium iodide staining (Krishan, 1975; Deitch et al., 1982). Actual tumour cell diameters were determined by direct measurements of 100 tumour cells per cell line using an inverted microscope equipped with a reticle and an internal standard. The results are expressed as the mean cell diameter and the standard deviation.

*Chromosome analysis*

Cultures were harvested for karyotypic analysis, slides prepared and G-banding performed as previously described (Trent & Thompson, 1987). A minimum of 30 cells per cell line were analysed, with results expressed according to ISCN recommendations (Harden & Klinger, 1985). Karyotypic information on the parental MCF7 cell line has been presented previously (Osborn et al., 1987).

*Drug accumulation*

Cellular accumulation of drug was determined during a 1 h exposure of an aliquot of cells ($1 \times 10^{6}$ cells plated 2 days previously into a 35 mm$^{2}$ petri dish) to $^{14}$C DOX-5.0 $\mu$M, $^{14}$C Mitox-10.0 $\mu$M, and $^{3}$H VCR-1.0 $\mu$M at 37°C. These drug concentrations were chosen based on the relative specific activities of the three radiolabelled agents in an attempt to achieve similar counts per minute (c.p.m.) in MCF7/S cells. One hour after the addition of radiolabelled drug, the cells were washed twice with ice cold phosphate buffered saline (PBS) trypsinised, incubated with 0.2 N NaOH for 2 h and neutralised with 0.2 N HCl (equivalent volumes). The amount of radiolabel (c.p.m.) was determined using liquid scintillation counting. Each experiment was repeated a minimum of three times and data from a representative experiment are shown.

For all experiments involving VER, cells were incubated in media containing 6 $\mu$g ml$^{-1}$ (13.2 $\mu$M) VER for 15 min prior to drug exposure and during the 1 h drug exposure.

*Mitoxantrone efflux studies*

Cells were incubated with $^{14}$C Mitox for 1 h as described in the Drug Accumulation section. At the end of 1 h, cells were placed on ice and washed once with ice cold PBS; drug-free RPMI complete media (2% foetal bovine serum) was added, and cells were incubated at 37°C for up to 60 min. At the end of each time period, cells were washed, trypsinised, digested, and counted as described previously. Efflux at each time point was determined by:

$$\frac{\text{c.p.m. } ^{14}\text{C Mitox}}{\text{c.p.m. } ^{14}\text{C Mitox at time 0}} \times 100$$

Each experiment was repeated a minimum of three times and data from a representative experiment are shown.

*Non-protein sulfhydryl (NPSH) measurements*

The NPSH content of the MCF7 cell lines was measured using the method of Sedlak and Lindsay (1968). A total of $5 \times 10^{6}$ cells was washed twice with iced PBS (pH 7.4) and transfered to a microcentrifuge tube where they were lysed by sonication (model 250 Branson sonifier, Danbury, CT). Cellular protein was precipitated by addition of 5% sulfosalicylic acid. The cell lysate was then centrifuged at 12,000 *g* for 5 min at room temperature and a 1 ml aliquot of the supernatant was transferred to a tube containing 0.2 M Tris buffer (pH 8.9). To each tube, 100 $\mu$l of 0.01 N 5,5′-dithiobis(2-nitrobenzoic acid) in absolute methanol was added. The contents were mixed and the absorbance of each sample was measured at 412 nm. The concentration of NPSH in the sample was determined by comparing the optical density reading of the sample to a standard curve constructed using reduced glutathione. NPSH levels were analysed 4 days post cell passage in all lines. Protein measurements were determined according to the method of Lowry et al. (1951).

*Immunoblot analysis for P-glycoprotein*

Plasma membranes were purified from $2 \times 10^{8}$ cells (Riordan & Ling, 1979). Polyacrylamide gel electrophoresis (50 $\mu$g protein per lane) was performed according to the method of Laemmli (1970). Protein was transferred from sodium dodecyl sulphate-polyacrylamide gels onto nitrocellulose filter paper according to the method of Towbin *et al.* (1979). The nitrocellulose filters were then incubated overnight at room temperature in tris-PBS (TPBS) containing the C219 monoclonal antibody (IgG) (kindly provided by Dr Victor Ling, Ontario Cancer Institute, Toronto, Ontario, Canada) at a concentration of 10 ng $ml^{-1}$ (Kartner *et al.*, 1985). Following wash steps to remove unbound antibody, the filters were incubated overnight at room temperature in 50 ml of TPBS containing 25 μg of $^{125}$I-labelled rabbit anti-mouse IgG (specific activity – 600 μCi $ml^{-1}$, New England Nuclear, Boston, MA). The filters were again washed to remove the unbound secondary antibody, dried, and exposed to X-Omat AR film (Kodak). A membrane preparation from 8226/DOX40 multiple myeloma cells was added as a positive control for P-glycoprotein (Dalton *et al.*, 1986).

### *RNA analysis*

RNA was isolated from MCF7 cells using guanidium isothiocynate and cesium chloride centrifugation (Maniatis *et al.*, 1982). Slot blot analysis of total cellular RNA was carried out as described (Davis *et al.*, 1986) using a 640 bp cDNA fragment of mdr-1, p-CHP-1, isolated from a colchicine-resistant CHO cDNA library (Riordan *et al.*, 1985). The probe was oligolabelled according to the method of Feinberg and Vogelstein (1983). Human multiple myeloma cells displaying the multidrug resistant phenotype (8226/DOX40) were included as positive controls and the drug sensitive parent cells (8226/S) as negative controls. The blots were also probed with $^{32}$P-labelled human tubulin cDNA (American Type Culture Collection, Rockville, MD) to confirm the amount of RNA contained in each sample.

### *Statistical methods*

The *P*-values for the comparison of drug accumulation with and without VER were calculated using a two-sample independent *t*-test. The *P*-values for the difference in drug accumulation between cell lines were calculated using Tukey's Studentised Range Test and the Bonferroni multiple comparisons procedure (Miller, 1981). The 95% confidence limits of the doubling times were determined from the slope of the linear portion of the growth curves (Mood *et al.*, 1974). The slopes of the Mitox efflux curves were evaluated by Analysis of Covariance (Snedecor & Cochran, 1980).

### Results

### *Growth characteristics*

The growth characteristics of the sensitive, parental line (MCF7/S), DOX resistant variant (MCF7/D40) and Mitox resistant variant (MCF7/Mitox) are listed in Table I. The doubling times for MCF7/S, MCF7/D40 and MCF7/Mitox cells were similar. No differences between the fraction of cells in S-phase were demonstrated between the three cell lines. The mean cell diameter varied slightly among the three cell lines, but the confidence intervals (standard deviation) were overlapping.

### *Dose response curves*

Dose response curves for MCF7/S and MCF7/D40 cells after exposure to DOX (with and without VER) as determined by the MTT assay are depicted in Figure 1a. Similar curves for MCF7/Mitox cells after exposure to Mitox (with and without VER) are depicted in Figure 1b. Significant resistance to DOX and Mitox was observed for both MCF7/D40 and MCF7/Mitox cells, respectively. However, the addition of

**Figure 1** Dose response of MCF7/S (●) and MCF7/D40 (■) cells to DOX alone (close symbols) and in combination with VER (open symbols) (VER-6 μg$ml^{-1}$, 13.2 μM, MTT assay) (**a**). Dose response of MCF7/S (●) and MCF7/Mitox (■) cells to Mitox alone (closed symbols) and in combination with VER (open symbols) (VER-6 μg $ml^{-1}$, 13.2 μM, MTT assay) (**b**). The data are presented as the per cent of control optical density (o.d.).

**Table I** Growth characteristics

| Characteristic | MCF7/S | Cell line MCF7/D40 | MCF7/Mitox |
|---|---|---|---|
| Doubling time, hours (95% confidence limits) | 41.6 (32.1–59.0) | 35.2 (25.5–56.8) | 40.3 (35.0–47.6) |
| [a]Fraction of cells in S-phase | 38% | 38% | 31% |
| [b]Relative cell size | 1.00 | 1.09 | 1.00 |
| Mean cell diameter in microns (SD) | 18.5 (3.5) | 21.0 (6.1) | 17.5 (3.0) |

[a]Determined using propidium iodide and flow cytometry. [b]Determined using flow cytometry, MCF7/S normalised to 1.00.

VER affected resistance only for the MCF7/D40 cell line. VER alone was minimally toxic to the cells (data not shown). The 50% inhibitory concentration ($IC_{50}$) of DOX (with and without VER) against MCF7/D40 cells and the $IC_{50}$ of Mitox (with and without VER) against MCF7/Mitox cells are shown in Table II. With the addition of VER the $IC_{50}$ of DOX against MCF7/D40 cells decreased 10-fold (30 μM vs 3.1 μM). In contrast, the $IC_{50}$ of Mitox against MCF7/Mitox cells was essentially unchanged with VER exposure (64 μM vs 91 μM). The experiments performed with the MTT assay were repeated using a clonogenic assay (Malinin & Perry, 1967) for Mitox with and without VER in all three cell lines (Table II). The $IC_{50}$s from the clonogenic assays were generally lower than those obtained from the MTT assays. However, the relative degree of resistance between the cell lines was similar. In addition, the $IC_{50}$ from the clonogenic assay for Mitox against MCF7/Mitox cells remains unchanged with VER exposure (7.7 μM vs 7.4 μM).

### Cross resistance patterns

Table II demonstrates the sensitivities of the 3 MCF7 cell lines to a number of chemotherapeutic agents. The data are expressed as the $IC_{50}$ and as the relative amount of resistance of MCF7/D40 and MCF7/Mitox cells to MCF7/S cells ($IC_{50}$ ratio). A high degree (75 fold) of resistance to DOX was observed in MCF7/D40 cells. In addition, MCF7/D40 cells were cross resistant to a number of other drugs: VCR (190 fold), Mitox (153 fold), VLB (93 fold), m-AMSA (41 fold). The addition of VER partially reversed the resistance of MCF7/D40 cells to DOX, VCR and Mitox.

MCF7/Mitox cells displayed a very high degree of resistance to Mitox (1208 fold) which was not reversed by VER. A lesser degree of cross resistance to DOX (8.3 fold) and VCR (22 fold) was observed. VER was ineffective in reversing resistance to both DOX and VCR in MCF7/Mitox cells. The MCF7/Mitox cell line was cross resistant to VLB (43 fold), m-AMSA (42 fold) VP-16 (26 fold) and CDDP (21.5 fold). Only low levels of resistance to L-PAM, MitoC, Gram D or 5FU were seen in either MCF7/D40 or MCF7/Mitox cells.

### Drug accumulation studies

The 1 h net intracellular accumlations of $^{14}C$ DOX, $^{3}H$ VCR and $^{14}C$ Mitox are displayed in Figure 2. In both the MCF7/D40 and MCF7/Mitox cell line, accumulation of $^{14}C$ DOX (Figure 2a), $^{3}H$ VCR (Figure 2b) and $^{14}C$ Mitox (Figure 2c) was decreased relative to the MCF7/S cell line. The MCF7/Mitox cell line had less net intracellular drug accumulation for DOX and Mitox compared to the MCF7/D40 cell line ($P<0.05$ for DOX and Mitox) and the MCF7/S parent cell line ($P<0.05$ for DOX and Mitox). Both resistant cell lines had decreased VCR net intracellular accumulation relative to MCF7/S ($P<0.05$ for MCF7/D40 and MCF7/Mitox) but were not different from each other ($P>0.05$).

The effects of VER in increasing DOX and VCR intracellular drug accumulation were limited to the MCF7/D40 resistant cell line (41% and 298% increased, respectively) with no effect observed in the MCF7/S or MCF7/Mitox cell lines. A slight but significant increase in Mitox accumulation was seen in both the MCF7/S (25%) and MCF7/Mitox (24%) cell lines with a greater increase seen in the MCF7/D40 cell line (63%).

Curves of Mitox efflux versus time in minutes for MCF7/S, MCF7/D40 and MCF7/Mitox cells were depicted in Figure 3. At each time point tested, relatively less drug persisted in MCF7/Mitox and MCF7/D40 cells as compared with MCF7/S cells. At the 5 min time point MCF7/D40 and MCF7/Mitox cells contained 34% (2149 c.p.m.) and 24%

**Table II** Cross resistance patterns

| Drug | MCF7/S | $IC_{50}{}^{a}$, μM ($IC_{50}$ratio[b]) MCF7/D40 | MCF7/Mitox |
|---|---|---|---|
| DOX[c] | 0.4 | 30 | 3.3 |
| | | (75) | (8.3) |
| DOX + VER | 0.37 | 3.1 | 3.5 |
| | | (8.4) | (9.5) |
| VCR | 0.0039 | 0.74 | 0.086 |
| | | (190) | (22) |
| VCR + VER | 0.0031 | 0.065 | 0.16 |
| | | (21) | (52) |
| Mitox | 0.053 | 8.1 | 64 |
| | | (153) | (1208) |
| | [0.011][d] | [0.14] | [7.7] |
| Mitox + VER | 0.053 | 1.2 | 91 |
| | | (23) | (1717) |
| | [0.0071] | [0.034] | [7.4] |
| VLB | 0.0070 | 0.65 | 0.30 |
| | | (93) | (43) |
| CDDP | 1.3 | 6 | 28 |
| | | (4.6) | (21.5) |
| VP-16 | 2.9 | 33 | 76 |
| | | (11) | (26) |
| m-AMSA | 0.86 | 35 | 36 |
| | | (41) | (42) |
| L-PAM | 16 | 40 | 91.4 |
| | | (2.5) | (5.7) |
| MitoC | 0.72 | 5.3 | 6.3 |
| | | (7.4) | (8.8) |
| Gram D | 0.15 μg ml$^{-1}$ | 1.2 μg ml$^{-1}$ | 0.98 μg ml$^{-1}$ |
| | | (8) | (6.5) |
| 5FU | 30 | 103 | 117 |
| | | (3.4) | (3.9) |

[a]50% Inhibitory Concentration, as determined by MTT assay; [b]Ratio of $IC_{50}$ for the drug resistant cell line to MCF7/S; [c]Abbreviations:

| | |
|---|---|
| DOX = Doxorubicin | VP-16 = Etoposide |
| VCR = Vincristine | m-AMSA = Amsacrine |
| VER = Verapamil | L-PAM = Melphalan |
| Mitox = Mitoxantrone | MitoC = Mictomycin C |
| VLB = Vinblastine | Gram D = Gramacidin D |
| CDDP = Cisplatinum | 5FU = 5-Fluorouracil; |

[d][] = $IC_{50}$ as determined by clonogenic assay.

**Figure 2** One hour net accumulation of $^{14}C$ DOX (5.0 μM), $^{3}H$ VCR (1.0 μM) and $^{14}C$ Mitox (10.0 μM) in MCF7/S, MCF7/D40 and MCF7/Mitox cells with (closed bars) and without (open bars) VER (6 μg/ml$^{-1}$), 13.2 μM). Error bars indicate the standard error of the mean with replicates of three. P-values for the effect of VER are indicated above the respective open and closed bars. The decreased accumulation of each of the three drugs alone in MCF7/D40 and MCF7/Mitox cells relative to MCF7/S cells were statistically significant ($P<0.05$ in each instance).

**Figure 3** Efflux of Mitox in MCF7/S (●), MCF7/D40 (▲) and MCF7/Mitox (■) cells versus time (minutes). Data points are expressed as per cent of $^{14}C$ Mitox present at time 0. Error bars indicate the standard error of the mean with replicates of three.

(1526 c.p.m.) respectively, of the amount of drug contained in MCF7/S cells (6299 c.p.m.). These values were statistically significant at $P<0.05$ for MCF7/S cells compared to both MCF7/D40 and MCF7/Mitox cells. Furthermore, in MCF7/Mitox and MCF7/D40 cells drug efflux was more rapide during the initial 5 min as compared to MCF7/S cells. Statistical comparison of the slopes of the efflux curves during this time period revealed $P = 0.055$ and 0.0063 for MCF7/S cells compared to MCF7/D40 and MCF7/Mitox cells, respectively. At time points beyond 10 min the slopes of the efflux curves were not significantly different.

### P-*glycoprotein detection*

An immunoblot probed for the presence of *P*-glycoprotein using the C219 antibody is seen in Figure 4. The 8226/DOX40 cell line was included as a positive control for *P*-glycoprotein (Dalton *et al.*, 1986). *P*-glycoprotein was detected in the MCF7/D40 cell line but was not seen in the MCF7/S and MCF7/Mitox cell lines.

### *RNA analysis*

Slot blot analysis revealed no evidence of mdr-1 message in the MCF7/S or MCF7/Mitox cell lines but was found to be expressed in the MCF7/D40 line thus confirming our findings using western blotting (Figure 5). Thus, selection with the anthracycline DOX resulted in an expression of the *P*-glycoprotein message while selection with the antracenedione mitoxantrone did not.

**Figure 4** Immunoblot for the presence of *P*-glycoprotein (C219 antibody) in MCF7/S, MCF7/Mitox and MCF7/D40 cells (8226/DOX40 cell line – positive control).

### *Cytogenetic studies*

Chromosomal analysis of the MCF7 parent cell line and its DOX and Mitox resistant sublines were performed using G-banding analysis. Detailed cytogenetic analysis of the parental line has been published previously (Osborne *et al.*, 1987). There were obvious similarities between all three cell lines clearly indicating their common origin. All three cell lines demonstrated a near-triploid chromosome number (72–80) with numerous structural and numeric alterations (Table III). The MCF7/D40 cell line deviated the most significantly from the parental cell line (primarily by the addition of new marker chromosomes). The MCF7/Mitox cell line more closely resembled the parental line with the only differences being a loss of the marker chromosomes 1p−, 6q−, 7pHSR, 13q+, 16q+ and the addition of one marker unique to this cell line (3p+). The parental cell line (which is known to be amplified for N-ras) (Graham *et al.*, 1985) displayed cytologic evidence of gene amplification in the form of a homogeneous staining region. This marker was lost in both resistant sublines and neither displayed double minutes or other HSRs.

**Figure 5** Slot blot analysis of MCF7 RNA. Serial two-fold dilutions of total cellular RNA (10, 5, 2.5, 1.25, 0.6, 0.3 μg, left to right) were applied to the filter. After hybridisation with the p-CHP-1 cDNA probe, the filter was exposed for 24 h with an intensifying screen at −80°C.

**Table III** Cytogenetic analysis of MCF7 parental and resistant sublines

| *Marker* | *MCF7/S* | *MCF7/D40* | *MCF7/Mitox* |
|---|---|---|---|
| 1p− | + | | |
| 2q+ | + | | + |
| der(2) | | + | |
| 2q− | | + | |
| 3p− | + | + | + |
| 3p+ | | | + |
| 5p+ | + | + | + |
| 6p+ | | + | |
| 6q+ | + | + | + |
| 6q− | + | + | |
| 7p+ | + | | + |
| 7pHSR | + | | |
| inv(7) | + | + | + |
| 9p+ | | + | |
| 12q− | + | + | + |
| 13q+ | + | + | |
| 16q+ | + | | |
| 17q+ | | + | |
| t(12;19) | + | + | + |
| t(22;22) | | + | |
| Xq− | + | | + |
| Xq+ | | + | |
| t(X;?) | | + | |

*Non-protein sulfhydryl (NPSH) measurements*

The MCF7/S cell line was found to have significantly elevated amounts of NPSH compared to the other two cell lines ($25.43 \pm 0.34$ nmol $10^{-6}$ cells; $P < 0.001$; Student's *t*-test). NPSH levels in MCF7/D40 and MCF7/Mitox cells were decreased at $16.23 \pm 0.28$ and $15.83 \pm 0.21$ nmol $10^{-6}$ cells respectively (not significant, Student's *t*-test). Total protein measurements revealed no differences among the three cell lines (data not shown).

### Discussion

In this report we describe the development of two multidrug-resistant cell lines established from a common parental cell line using DOX and Mitox as selecting agents. Of note is the fact that Mitox resistance developed much quicker (6 months) than DOX resistance (greater than 2 years). In establishing the drug resistant variants the concentrations of the selecting agents were increased as rapidly as allowed by cell growth. Indeed, the concentration of DOX could be increased only very slowly in the selection of the MCF7/DOX cell line. However, in spite of a different degree of selection pressure for the MCF7/Mitox cell line (lower final Mitox concentration, shorter selection time) relative to the MCF7/DOX cell line, a greater degree of Mitox resistance (1208 fold *vs* 75 fold) developed. Formal fluctuation analysis tests were not performed and the rate of mutation to resistance for the individual drugs cannot be stated.

The DOX resistant (MCF7/D40) and Mitox resistant (MCF7/Mitox) cell lines have both similarities and differences in cross resistance to other agents. A broad range of drugs with varied mechanisms of action were studied: DNA binding/intercalating agents (DOX, Mitox), tubulin binding agents (VCR, VLB), alkylating agents (L-PAM, CDDP, Mito-C) topoisomerase II inhibitors (DOX, Mitox, VP-16, m-AMSA), an antimetabolite (5 FU) and a cell membrane ionophore (Gram-D). In addition to being resistant to DOX, MCF7/D40 cells were highly resistant to the tubulin binding agents, moderately resistant to the topoisomerase agents and displayed lower levels of resistance to the alkylating agents and the antimetabolite 5 FU. Cross-resistance to Mitox was also high in the MCF7/D40 cell line. The MCF7/Mitox cell line displayed a number of unique characteristics. It developed a very high degree of resistance (1208 fold) to the primary selecting agent (Mitox) with relatively minor cross resistance to DOX (8.3 fold) and VCR (22 fold). In addition, MCF7/Mitox cells were partially cross resistant to CDDP, an agent not commonly associated with the multidrug-resistance phenotype (21.5 fold resistant).

Other Mitox resistant cell lines have been reported and are similar to the Mitox resistant MCF7 cell line in that *P*-glycoprotein is not over-expressed. Wallace *et al.* first reported a human colon carcinoma cell line selected for resistance to Mitox (WiDr) (Wallace *et al.*, 1987). This line was further characterised by Dalton *et al.* demonstrating decreased intracellular accumulation of both Mitox and DOX with no increased expression of *P*-glycoprotein (Dalton *et al.*, 1988). Harker *et al.* recently reported an HL-60 leukaemic cell line selected for resistance to Mitox which also lacked *P*-glycoprotein overexpression (Harker *et al.*, 1989).

Marsh *et al.* reported a multi-drug resistant HL-60 cell line which over-expressed a 150,000 dalton membrane protein distinct from *P*-glycoprotein (Marsh & Center, 1988). These investigators felt that the P150 protein was involved in the resistance mechanism and contributed to the decreased intracellular drug accumulation. Whether the Mitox resistant cell lines over-express a novel drug-resistance related membrane protein remains to be determined. Studies are currently in progress to further describe the mechanism of enhanced drug efflux in the MCF7/Mitox cell line.

Other drug resistance mechanisms unrelated to drug transport may be important in conferring drug resistance. A number of 'atypical' MDR cell lines have recently been reported. Danks *et al.* reported a leukaemic cell line (CEM/VM-1) selected for resistance to the epipodophyllotoxin, VM-26 (Danks *et al.*, 1987). This cell line was felt to display 'atypical' MDR because it was not cross resistant to Vinca alkaloids and did not display a decreased cellular accumulation of drug. Slovak *et al.* reported two DOX resistant cell lines which had different mechanisms of resistance; one associated with *P*-glycoprotein, the other more atypical in its mechanism of resistance (Slovak *et al.*, 1988). Mirski *et al.* reported a DOX resistant human small cell lung cancer cell line (Mirski *et al.*, 1987) which displayed a typical cross resistance pattern to VP-16, Vinca alkaloids and colchicine but did not over-express *P*-glycoprotein.

In summary, this study demonstrates that two compounds (DOX and Mitox) with structural similarities result in different mechanisms of resistance when used as selecting agents in the same human breast cancer cell line. Reduced drug accumulation secondary to enhanced drug efflux accounts, at least in part, for the multidrug resistant phenotype in both resistant cell lines. However, only the DOX resistant cell line over-expresses *P*-glycoprotein compared to the drug sensitive parent cell line. It remains to be determined why DOX, a natural product, induces *P*-glycoprotein mediated MDR; whereas, Mitox, a synthetic compound induces a different mechanism of MDR.

The authors would like to thank Judith Gooley for her technical assistance and B. Kathryn Monroe for typing the manuscript. This work was supported in part by National Cancer Institute Grants CA17094, CA43043 and CA41183. C.W.T. was a recipient of a Clinical Oncology Career Development Award from the American Cancer Society.

### References

ALBERTS, D.S., GRIFFITH, K.S., GOODMAN, G.E., HERMAN, T.S. & MURRAY, E. (1980). Phase I clinical trial of mitoxantrone: a new anthracenedione anticancer drug. *Cancer Chemother. Pharmacol.*, **5**, 11.

BATIST, G., TULPULE, A., SINHA, B.K., KATKI, A.G., MYERS, C.E. & COWAN, K.H. (1986). Overexpression of a novel anionic glutathione transferase in multidrug-resistant human breast cancer cells. *J. Biol. Chem.*, **261**, 15544.

CARMICHAEL, J., DEGRAFF, W.G., GAZDAR, A.F., MINNA, J.D. & MITCHELL, J.B. (1987). Evaluation of a tetrazolium-based semiautomated colorimetric assay: Assessment of chemosensitivity testing. *Cancer Res.*, **47**, 936.

COWAN, K.H., BATIST, G., TULPULE, A., SINHA, B.K. & MYERS, C.E. (1986). Similar biochemical changes associated with multidrug resistance in human breast cancer cells and carcinogen-induced resistance to zenobiotics in rats. *Proc. Natl Acad. Sci. USA*, **83**, 9328.

DALTON, W.S., CRESS, A.E., ALBERTS, D.S. & TRENT, J.M. (1988). Cytogenetic and phenotypic analysis of a human colon carcinoma cell line resistant to mitoxantrone. *Cancer Res.*, **48**, 1882.

DALTON, W.S., DURIE, B.G.M., ALBERTS, D.S., GERLACH, J.H. & CRESS, A.E. (1986). Characterization of a new drug-resistant human myeloma cell line that expresses *P*-glycoprotein. *Cancer Res.*, **46**, 5125.

DALTON, W.S. (1991). Management of systemic matastases and the sequential therapy of advanced disease. In: Bland, K.I. & Copeland, E.M. (eds). *The Breast: A Comprehensive Textbook for the Management of Benign and Malignant Diseases*, pp. 877–899. W.B. Saunders Company.

DANKS, M.K., YALOWICH, J.C. & BECK, W.T. (1987). Atypical multiple drug resistance in a human leukemic cell line selected for resistance to teniposide (VM-26). *Cancer Res.*, **47**, 1297.

DAVIS, L.G., DIBNER, M.O. & BATTEY, J.F. (1986). Preparation and analysis of RNA from eukaryotic cells. In *Basic Methods in Molecular Biology*. p. 129. Elsevier: New York.

DEITCH, A.D., LAW, H. & WHITE, R.D. (1982). A stable propidium iodide staining procedure for flow cytometry. *J. Histochem. Cytochem.*, **30**, 967.

DENIZOT, F. & LANG, R. (1986). Rapid colorimetric assay for cell growth and survival: modifications to the tetrazolium dye procedure giving improved sensitivity and reliability. *J. Immunol. Methods*, **89**, 271.

FEINBERG, A.P. & VOGELSTEIN, B. (1983). A technique for radiolabeling DNA restriction endonuclease fragments to high specific activity. *Anal. Biochem.*, **132**, 6.

FUQUA, S.A.W., MORETTI-ROJAS, I.M., SHNEIDER, S.L. & MCGUIRE, W.L. (1987). *P*-glycoprotein expression in human breast cancer cells. *Cancer Res.*, **47**, 2103.

GERLACH, J.H., KARTNER, N., BELL, D.R. & LING, V. (1986). Multidrug resistance. *Cancer Surveys*, **5**, 25.

GRAHAM, K., RICHARDSON, C., MINDEN, M., TRENT, J. & BUICK, R. (1985). Varying degress of amplification of the N-*ras* oncogene in the human breast cancer cell line MCF-7. *Cancer Res*, **45**, 2201.

HARDEN, D.G. & KLINGER, H.P. (1985). International System for Human Cytogenetic Nomenclature (ISCN). In *Cytogenet and Cell Genet.*, Jensen, J.T. & Kaelbing, M. (eds). **21**, 1.

HARKER, W.G., SLADE, D.L., DALTON, W.S., MELTZER, P.S. & TRENT, J.M. (1989). Multidrug resistance in mitoxantrone-selected HL-60 leukaemia cells in the absence of *P*-glycoprotein overexpression. *Cancer Res.*, **49**, 4542.

KARTNER, N., EVBERNDEN-PORELLE, D., BRADLEY, G. & LING, V. (1985). Detection of *P*-glycoprotein in multidrug-resistant cell lines by monoclonal antibodies. *Nature*, **316**, 820.

KRISHAN, A. (1975). Rapid flow cytofluorometric analysis of mammalian cell cycle by propidium iodide staining. *J. Cell. Biol.*, **66**, 188.

LAEMMLI, U.K. (1970). Cleavage of structural proteins during the assembly of the head of bacteriophage T4. *Nature*, **227**, 680.

LOWRY, O.H., ROSENBROUGH, N.J., FARR, A.L. & RANDALL, R.J. (1951). Protein measurements with the folin phenol reagent. *J. Biol. Chem.*, **193**, 265.

MALININ, T.I. & PERRY, V.P. (1967). A review of tissue culture and organ viability assay. *Cryobiology*, **4**, 104.

MANIATIS, T., FRITSCH, E.F. & SAMBROOK, J. (1982). Extraction, purification, and analysis of mRNA from eukaryotic cells. In: *Molecular Cloning: A Laboratory Manual*. Sambrook, J. (ed.) p. 187. York: Cold Spring Harbor: New York.

MARSH, T. & CENTER, M.S. (1988). Mechanism of multidrug resistance in HL60 cells: evidence that a surface membrane protein distinct from *P*-glycoprotein contributes to reduced cellular accumulation of drug. *Cancer Res.*, **48**, 3959.

MILLER, R.G. (1981). Normal univariate techniques. In: *Simultaneous Statistical Inference*, p. 37. Springer-Verlag: New York.

MIRSKI, S.E.L., GERLACH, J.H. & COLE, S.P.L. (1987). Multidrug resistance in a human small cell lung cancer cell line selected in Adriamycin. *Cancer Res.*, **47**, 2594.

MOOD, A.M., GRAYBILL, F.A. & BOES, D.C. (1974). Parametric interval estimation. In: *Introduction to the Theory of Statistics*, p. 378. McGraw-Hill: New York.

OSBORNE, K., HOBBS, K. & TRENT, J. (1987). Biological differences among MCF-7 human breast cancer cell lines from different laboratories. *Breast Cancer Res & Treat.*, **9**, 111.

PASTAN, I. & GOTTESMAN, M. (1987). Multiple-drug resistance in human cancer. *N. Engl. J. Med.*, **316**, 1388.

RIORDAN, J.R. & LING, V. (1979). Purification of *P*-glycoprotein from plasma membrane vesicles of Chinese hamster ovary cell mutants with reduced colchicine permeability. *J. Biol. Chem.*, **254**, 12701.

RIORDAN, J., DEUCHARS, K., KARTNEW, N., ALON, N., TRENT, J. & LING, V. (1985). Amplification of *P*-glycoprotein genes in multidrug resistant mammalian cell lines. *Nature*, **316**, 817.

SEDLAK, J. & LINDSAY, R.H. (1968). Estimation of total, protein-bound, and nonprotein sulfhydryl groups in tissue with Ellman's reagent. *Anal. Biochem.*, **25**, 192.

SHENKENBERG, T.D. & VON HOFF, D.D. (1986). Mitoxantrone: a new anticancer drug with significant clinical activity. *Ann. Int. Med.*, **105**, 67.

SLOVAK, M.L., HOELTGE, G.A., DALTON, W.S. & TRENT, J.M. (1988). Pharmacological and biological evidence for differing mechanisms of doxorubicin resistance in two human tumor cell lines. *Cancer Res.*, **48**, 2793.

SMYTH, J.F., CORNBLEET, M.A., STUART-HARRIS, R.C. & 7 others (1984). Mitoxantrone as first-line chemotherapy for advanced breast cancer: Results of a European collaborative study. *Sem. Oncol.*, **11**, 15.

SNEDECOR, G.W. & COCHRAN, W.G. (1980). In: *Statistical Methods*. The Iowa State University Press: Ames, Iowa.

TORMEY, D.C. (1975). Adriamycin (NSC-123127) in breast cancer: An overview of studies. *Cancer Chemother. Rep.*, **6**, 319.

TOWBIN, H., STAEHELIN, T. & GORDON, J. (1979). Electrophoretic transfer of proteins from polyacrylamide gels to nitrocellulose sheets: procedure and some applications. *Proc. Natl Acad. Sci. USA*, **76**, 4350.

TRENT, J.M. & THOMPSON, F.M. (1987). Methods for chromosome banding of human and experimental tumors *in vitro*. In: *Methods in Enzymology*. Gottesman, M. (ed.) p. 267. Academic Press: New York.

WALLACE, R.E., LINDH, D. & DURR, F.E. (1987). Development of resistance and characteristics of a human colon carcinoma subline resistant to mitoxantrone *in vitro*. *Cancer Inv.*, **5**, 417.

YAP, H.Y., BLUMENSCHEIN, G.R., SCHELL, F.C., BUZDAR, A.U., VALDIVIESO, M. & BODEY, G.P. (1981). Dihydroxyanthracenedione: a promising new drug in the treatment of metastatic breast cancer. *Ann. Int. Med.*, **95**, 694.